# Strategic roadmap for delivery of clinical trials in rare childhood central nervous system (CNS) tumours: a multi-stakeholder consensus

**DOI:** 10.1038/s41416-026-03589-6

**Published:** 2026-08-22

**Authors:** Jenny Adamski, John R. Apps, Sarita Depani, Rebecca M. Hill, Barry Pizer, Thankamma Ajithkumar, Simon Bailey, Kim S. Bull, Fernando Carceller, Julia V. Cockle, Robert A. Dineen, Jessica A. Douglas‑Pugh, Jenny Gains, Debbie Hicks, Mette Jorgensen, John-Paul Kilday, Juan-Pedro Martinez‑Barbera, Timothy A. Ritzmann, Emma Thompson, Simon Gates, Darren Hargrave, Allan Hackshaw, G. A. Amos Burke, Steven C. Clifford

**Affiliations:** 1 Birmingham Women’s and Children’s NHS Foundation Trust, Birmingham, UK; 2 Cancer and Genomic Sciences, University of Birmingham, Birmingham, UK; 3 Great Ormond Street Hospital for Children NHS Foundation Trust, London, UK; 4 Wolfson Childhood Cancer Research Centre, Newcastle University Centre for Cancer, Newcastle-upon-Tyne, UK; 5 University of Liverpool, Liverpool, UK; 6 Cambridge University Hospitals NHS Foundation Trust, Cambridge, UK; 7 University of Southampton, Southampton, UK; 8 The Royal Marsden NHS Foundation Trust, London, UK; 9 The Institute of Cancer Research (ICR), London, UK; 10 University of Nottingham, Nottingham, UK; 11 NIHR Nottingham Biomedical Research Centre, Nottingham, UK; 12 Cancer Research UK Clinical Trials Unit, School of Medical Sciences, College of Medicine and Health, University of Birmingham, Birmingham, UK; 13 University College London Hospitals NHS Foundation Trust (UCLH), London, UK; 14 Manchester University NHS Foundation Trust (MFT), Manchester, UK; 15 The Geoffrey Jefferson Brain Research Centre, University of Manchester, Manchester Academic Health Science Centre, Manchester, UK; 16 University College London (UCL) Great Ormond Street Institute of Child Health, London, UK; 17 Nottingham University Hospitals NHS Trust, Nottingham, UK; 18 The Brain Tumour Charity, Fleet, UK; 19 Cancer Research UK & UCL Cancer Trials Centre, UCL, London, UK

**Keywords:** Clinical trials, CNS cancer, Paediatric cancer

## Abstract

Rare paediatric central nervous system (CNS) tumours comprise a biologically-heterogeneous group of low-incidence diseases that present significant challenges to both therapeutic development and clinical research, limiting the availability of high-quality evidence to guide clinical care. The UK3CR Children’s Cancer Research Group (CRG) CNS Tumours Subgroup convened a multidisciplinary workshop to identify research priorities and establish a strategic framework for advancing clinical trials in this setting. Four high-priority tumour groups—craniopharyngioma, choroid plexus carcinoma (CPC), very high-risk medulloblastoma (VHR-MB), and rare embryonal and sarcomatous tumours (REST), including embryonal tumour with multilayered rosettes (ETMR)—were evaluated. Key outcomes included the feasibility of a UK-led craniopharyngioma trial, the need for enhanced national CPC data collection, and support for European early-phase platform trials in VHR-MB. REST highlighted transnational regulatory complexity requiring coordinated registries and parallel trial models. Cross-cutting barriers included regulatory discordance, contracting delays, fragmented funding, pharmacovigilance differences, data-sharing constraints, and interoperable infrastructures. The workshop emphasised innovative methodologies, including Bayesian/adaptive designs, platform trials, and external control arms, to maximise efficiency in small populations, alongside strengthened patient involvement and international collaboration. Collectively, these findings define a collaborative and internationally-aligned strategic roadmap to accelerate clinical research and improve outcomes in rare paediatric CNS tumours.

## Introduction

Rare central nervous system (CNS) tumours in children and young people represent one of the most complex, heterogeneous, and understudied areas within paediatric oncology. Although definitions are not standardised, it has been suggested ‘very rare’ childhood cancers are those with an incidence of less than 2 per million per year [1–3]. This broadly aligns with observations in childhood CNS tumours, where more common entities (e.g. low- and high-grade glioma, medulloblastoma, ependymoma) occur at rates of 1.5 to 5 per million per year [4] and are typically investigated through large, coordinated, randomised international clinical trials. In contrast, rarer cancer types such as choroid plexus carcinoma (CPC) and atypical teratoid rhabdoid tumours (ATRT) have incidences below 1.5 per 10 million per year, and the recent identification and sub-classification of novel molecularly-defined tumour classes (e.g. embryonal tumour with multilayered rosettes (ETMR; 3 per 100 million per year)) and sub-classes (e.g. very high-risk medulloblastoma (VHR-MB); 1 per 10 million per year) has led to an increasing number of well-defined ‘very-rare’ entities with distinct clinical needs [4–6].

Less common childhood CNS tumours may be more usefully defined by their strategic need—those where assembling investigational cohorts and conducting randomised trials has been challenging and/or limited by their incidence—and where a significant unmet need for clinical trials persists. For tumour types where few or no trials have been performed, treatment is often based on expert guidelines derived from extrapolation from other tumour types, cohort and registry studies, and case reports—and there is a need to improve the evidence base both for existing and novel treatments. Although individually uncommon, collectively these tumours constitute a significant proportion of paediatric neuro-oncology diagnoses and are associated with profound clinical, biological, regulatory, and infrastructural challenges. Clinical challenges include low cure rates (e.g. VHR-MB, ETMR, ATRT) and a significant burden of late effects in survivors.

Against this backdrop, the UK paediatric neuro-oncology community convened for a multi-disciplinary workshop to address how clinical trials for these rare tumour types with significant unmet need can be more effectively delivered and ensure that every patient contributes to shared learning.

The workshop had two overarching aims:

1. To review the current landscape of rare CNS tumour biology, unmet needs, and trial opportunities.
2. To identify concrete steps toward improving the feasibility, efficiency, and international alignment of clinical trials, especially those involving multi-national or transatlantic partnerships.

Across a full-day series of presentations, tumour-specific deep-dives, and cross-cutting trials methodology sessions, the workshop highlighted both the extraordinary scientific progress of recent years, and the major barriers that obstruct trial delivery. Critically, the meeting underscored the urgent need for unified transnational approaches integrating registries, contemporary trial designs, and creative approaches to biology rich investigation.

In this manuscript, we present a group consensus on major strategic themes impacting rare childhood CNS tumour research, alongside cross-cutting, disease-specific, organisational/operational and methodological opportunities to improve clinical research in these disease groups, culminating in recommendations for a coordinated strategy as part of this global effort.

## Workshop format and participants

The workshop, held in London in January 2026, was jointly facilitated by the UK Collaborative for Cancer Clinical Research (UK3CR) Children’s Cancer Research Group (CRG) CNS Tumours Subgroup (www.amrc.org.uk/uk-collaborative-for-cancer-clinical-research), and the Cancer Research UK Clinical Trials Units at the University of Birmingham (CRCTU) and University College London (Cancer Trials Centre, CTC), with contributions from twenty-four experts including trials unit Directors, statisticians specialising in advanced trial design, and experts across oncology, radiotherapy, radiology, endocrinology, late-effects and translational science. Contributors included early-career researchers (ECRs) and patient and public involvement and engagement (PPIE) partners. Discussions were structured around four rare CNS tumour groups requiring immediate coordinated action:

1. Craniopharyngioma
2. CPC
3. VHR-MB, namely *MYC*-amplified Group 3 and *MYCN*-amplified Sonic Hedgehog group molecular subclassifications (Group3-*MYC* and SHH-*MYCN*, respectively)
4. Rare embryonal and sarcomatous tumours (REST), including ETMR

Tumour types were selected for discussion to exemplify specific unmet clinical needs and distinct examples of major challenges in rare CNS tumour research. These include poor past records of clinical trials delivery and a need for morbidity-focused interventions (craniopharyngioma), very rare tumour types of either established pathology (CPC) or representing novel molecular classifications within historically clinically-defined groups (REST/ETMR), and rare very high-risk strata now recognised within more common tumour types (VHR-MB).

The programme integrated strategic presentations on UK–European–USA/North American trial delivery challenges, statistical design, disease-specific focussed sessions, and prioritisation of follow-up commitments to develop trial concepts, registries, and advisory structures, with a wider goal to establish key strategic principles for future trial delivery which apply across rare tumour types. A subsequent UK3CR Children’s CRG CNS Tumour Subgroup meeting in March 2026 reviewed outputs and refined next steps.

## Part one: challenges in the delivery of clinical trials for rare CNS tumours: the UK3CR subgroup perspective

The UK3CR Children’s CRG CNS Tumour Subgroup is a multi-disciplinary grouping of clinical and research experts responsible for the review and support of industry and academically-led clinical trials for childhood CNS tumours in the UK, alongside national coordination of best practice, collaborations and connections with strategic partners.

Clinical research in rare childhood CNS tumours faces a uniquely complex landscape defined by small patient populations, rapidly evolving tumour classifications, logistical barriers to international collaboration, and significant regulatory heterogeneity. Specific historical challenges include a lack of biologically-informed initiatives, underpowered trials, and the scarcity of novel therapeutics and accompanying early-phase trials.

The material presented in the workshop highlighted these multi-layered challenges encountered across the translational pathway from strategic planning and stakeholder engagement through trial design, governance, and operational delivery. Despite these challenges, UK trial sponsors, paediatric clinical networks and funding agencies have all shown continued commitment to these kinds of trials. Specific challenges highlighted were:

### Strategic and organisational challenges

Delivering rare CNS tumour trials often requires global reach which at present is most easily delivered by the pharmaceutical industry—who can act as a single global sponsor, have a legal presence in each country, and the funds and resources to complete all the regulatory activities. However, such rare or ultra-rare tumours may be of limited priority for large pharma and initiatives are typically driven by academic consortia and sponsors. These have been trying to develop a coordinated, national and international strategy involving clinical groups, funders, advocacy organisations, and research networks. International consortia have been developed to run early-phase trials in children, including the European Innovative Therapies for Children with Cancer (ITCC; www.itcc-consortium.org), the North American Paediatric Neuro-oncology Consortium (PNOC; www.pnoc.us) and the Collaborative Network for Neuro-oncology Clinical Trials (CONNECT; www.connectconsortium.org). Despite these consortia, trials in rare CNS tumours are few and typically focus on individual tumour types. Strategic discussions highlighted the need for improved integration, shared governance, and the creation of a stakeholder forum to align priorities and funding pathways. Enhanced engagement of ECRs, working groups across trial execution, imaging, drug delivery, and quality-of-survival research, and strengthened PPIE are foundational to this evolving ecosystem.

A recurring theme throughout discussions was the dependence of many proposed activities—including registries, biobanking, translational studies and international trials—on sustainable funding mechanisms. While infrastructure costs remain substantial, participants considered several recommendations realistic because they leverage existing national and international networks, registries and biobanking initiatives. Nonetheless, dedicated support from charitable funders, government agencies and industry partners will be essential to achieve long-term impact.

### Tumour-specific challenges and unmet clinical needs

Rare CNS tumours include high-grade and diffuse midline gliomas, medulloblastoma, ependymoma, ATRT, other embryonal tumour entities, germ cell tumours, craniopharyngioma, and CPC [4]. Each presents distinct unmet needs: lack of targetable options, fragmented trial landscapes, extremely low incidence, and in some cases, they have never been the subject of clinical trials or coordinated studies in the UK, despite substantial mortality and morbidity. Furthermore, the expansion of histology-agnostic precision-medicine trials may have limited molecular targets applicable to rare CNS tumour subtypes. Diverse pre-clinical evidence in appropriate model systems, and the relapse setting, remain particularly underserved; these data must be developed to a level of evidence necessary to support advancement to the clinic [7]. An additional strength within the UK is the development of disease-specific national clinical guidelines through groups such as the Children and Young People’s Cancer Association (CCLG; www.cclg.org) and disease-focused expert panels. These guidelines provide consensus standards of care, reduce variation in management, facilitate trial feasibility assessments, and provide a framework against which novel interventions can be evaluated.

### The need for trans-atlantic trials and why they are hard to deliver

Molecular subclassification has yet further splintered tumour groups into many ultra-rare entities, making global collaboration essential. Yet, attempts to deliver trans-Atlantic trials face consistent difficulties. Previous unpublished workshops involving ITCC and the US Children’s Oncology Group (COG) and Cancer Therapy Evaluation Programme (CTEP) have identified key barriers: funding misalignment; incompatible contract structures; divergent regulatory systems; data-protection conflicts between General Data Protection Regulation (GDPR) and North American requirements; concerns around intellectual property (IP) and data ownership. These challenges become most acute when trials involve unlicensed agents or carry future regulatory implications. A potential solution discussed at the workshop is the use of parallel sponsor models, whereby separate but harmonised North American and European studies operate under aligned protocols, eligibility criteria, endpoints and prospective data-sharing agreements. Such an approach may overcome regulatory and insurance barriers while preserving the scientific advantages of pooled analyses.

### Technical barriers: databases, GDPR, pharmacovigilance

Database integration remains one of the greatest challenges. European access to National Cancer Institute (NCI)/COG systems is hindered by investigator registration requirements, site assurances, and issues surrounding patient data transfer. Conversely, North American groups lack structures to function within European sponsor and national coordinating centre (NCC) models. Pharmacovigilance (PV) requirements differ substantially. The US Investigational New Drug (IND) definition is narrower than the UK/European Investigational Medicinal Products (IMP) definition, meaning some trials—considered low-risk in the US—require full IMP regulation in Europe. Adverse events reporting expectations also diverge, complicating harmonised oversight. Historical GDPR issues around “Right to Erasure” (or “Right to be Forgotten”) that allowed individuals to request the deletion of their personal data and impact for safety reporting have been resolved; under UK and EU GDPR, clinical trial data is now exempt from erasure, if deletion would seriously impair or render impossible the objectives of scientific research.

**Fig. 1:**
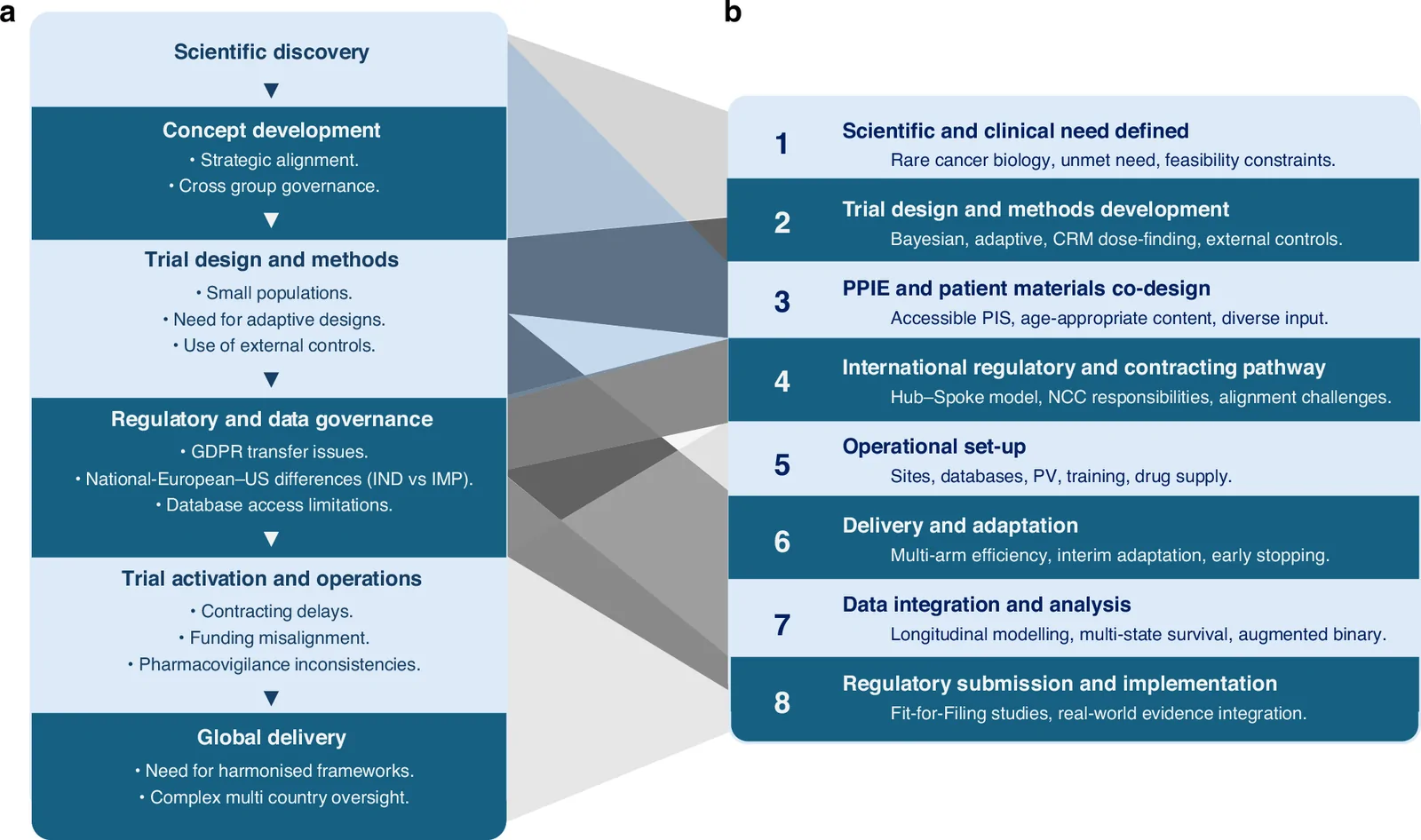
A translational pipeline for rare cancers. **a** Key challenges. (**b**) The rare disease trial lifecycle. CRM Continual reassessment model, GDPR General data protection regulation, IMP Investigational medicinal product, IND Investigational new drug, NCC National coordinating centre, PIS Patient information sheet, PV Pharmacovigilance.

A translational pipeline for rare cancers, with associated barriers, is presented in Fig. 1a and key challenges in rare childhood CNS tumours are summarised in Table 1A.

**Table 1:** A roadmap for delivery of high-quality trials in rare childhood CNS tumours.

A. Challenges
• Rare CNS tumour trials require global collaboration yet face major operational and regulatory obstacles • Many tumour variants and/or subtypes lack relapse trials, precision targets, or access to international studies • UK-European-N. American differences in operational delivery, GDPR, pharmacovigilance, and database standards, remain major blockers • Efficient, modern statistical designs are essential to maximise data from small populations • Early alignment across funders, regulators, CTUs, and industry is crucial to success
**B. Methodological Approaches**
• Rare cancer trials require efficient, rigorous, innovative designs • CRCTU and CTC deliver a broad portfolio including complex, early-phase, and regulatory-ready trials • International collaboration is essential but challenged by regulatory and operational differences • The Hub-and-Spoke model supports multinational delivery • Bayesian and adaptive methods maximise information use • PPIE is central to trial conceptualisation, funding and delivery • Registry data can aid understanding of current management and outcomes • Real-world evidence can strengthen rare-disease applications

A. Key challenges and B. Key methodological approaches to trials delivery.

## Part two: approaches to rare and paediatric cancer trials—the trials units’ perspective

There are two CRUK-core funded trials units in the UK that deliver most paediatric oncology trials: CRCTU (Birmingham) and CTC (UCL). They are leading national and international centres for the design and execution of high‑quality clinical trials across paediatric, adolescent, and adult oncology. Their mission rests on delivering innovative, biology‑rich, and patient‑centred trials capable of shaping clinical practice and supporting regulatory decision‑making. Together, these UK-based trials units currently coordinate or support 66 active paediatric-age clinical studies (46 at CRCTU and 20 at CTC), including both nationally led and internationally sponsored trials delivered through UK centres. The units demonstrate portfolio scale and diversity, and commitment to improving outcomes through scientifically rigorous and operationally efficient trial models. Key learnings and opportunities from this experience, and their relevance to rare childhood CNS tumours, were presented:

### Core capabilities and strategic themes

The two trials units conduct trials across early‑ and late‑phase development, including first‑in‑human trials, chemotherapy, immunotherapy, advanced therapy investigational medicinal products (ATIMPs), radiotherapy, surgical interventions, and stem cell transplantation. This can also include biomarker discovery and personalised medicine approaches. Both units provide the full-spectrum of support for investigators, covering protocol design, statistical input, regulatory submissions, pharmacovigilance (PV), data management, monitoring, and trial governance.

One of the key remits of the CRCTU is to conduct phase III paediatric oncology trials. A major focus is also the incorporation of complex innovative trial designs, particularly Bayesian adaptive methods, which enable efficient decision‑making even with small patient populations. The CRCTU now conducts “Fit for Filing” trials underscoring the ambition to design trials that can directly inform regulatory submissions [8]. The CTC has more focus on early phase paediatric trials with few centres, especially ATIMP trials (directly linked to a major research programme at UCL that generates new CAR-T products), advanced radiotherapy and medical devices that deliver systemic therapies, covering a range of cancer types: brain, neuroblastoma, sarcoma and haematological malignancies).

### International collaboration and brain tumour trial portfolio

The current landscape of global paediatric neuro‑oncology trials was highlighted, including international trials such as European Society for Paediatric Oncology (SIOPE)‑HRMB (Phase III; high-risk medulloblastoma), LOGGIC‑CORE (prospective registry; paediatric low-grade glioma), SIOPE-ATRT01 (prospective umbrella trial; ATRT), CONNECT TarGeT (prospective umbrella trial; HGG), BIOMEDE 2.0 (Phase III; diffuse midline glioma), CONNECT 2007 (Phase I/II; recurrent or progressive high-grade CNS tumours and meningiomas). Many of these are led from outside the UK and delivered nationally via one of the UK trials units, bringing significant challenges in trial set‑up and delivery. Despite this, international collaboration is necessary and achievable. Six major barriers to global paediatric trial delivery were identified: funding differences; regulatory misalignment; operational delivery complexity; variability in standards of care; differences in trial database systems; and cross‑border pharmaceutical supply constraints.

### Hub‑and‑spoke operating model

Both units apply a hub‑and‑spoke model in which responsibilities are shared between the International Major Sponsor and one of the UK trials units acting as the national coordinating centre, and which can work well. The UK unit handles national‑level regulatory submissions, site set‑up, oversight, patient information materials, data management, safety reporting, and trial committee coordination. But, while the UK can now readily implement trials sponsored by a US organization, the reverse is usually not true, mainly due to regulatory and insurance issues. Some adult oncology trials have overcome this by having two separately sponsored trials (US and elsewhere) but with a common protocol and key variables, and an agreement to analyse the trials together under a data sharing agreement.

### Roadblocks and strategic improvements

Key roadblocks in international trial delivery include a disparate understanding of regulatory frameworks, restricted access to USA/North American‑managed databases, data‑protection discrepancies, differences in PV rules drug release requirements, and different expectations towards PPIE. The trials units are currently addressing these through improved contracting processes, flexible regulatory strategies, strengthened relationships with PNOC, CONNECT and COG, and enhanced internal systems.

### Importance of PPIE

The involvement of patients and their parents and the public with relevant lived experience is an essential ethical component of rare disease trial design and conduct. But, it is important that PPIE representation is appropriate for a given tumour type. The aim is to ensure that the trial objectives are understood by patients and have clinical relevance, that the endpoints matter to patients, and whether participating in the trial is likely to be feasible, acceptable or burdensome. Given the age range for paediatric trials, age‑appropriate information materials are needed requiring several Patient Information Sheet (PIS) formats. For rare paediatric CNS tumours, developing national PPIE panels and using creative communication tools (e.g., simple PIS with many diagrams/graphics and well‑designed PIS videos) should improve recruitment and retention. Attendees noted examples of international PIS with many (~ 40) pages of heavy text being judged completely inaccessible by UK PPIE reviewers, requiring substantial revision and simplification. Attendees also noted from experience that significant PPIE (not just 1–2 patient representatives) is now a key requirement in grant applications to funders who wish to see evidence of early and meaningful patient partner engagement—from trial concept through to design—with plans to try to ensure diversity of patients.

The rare disease trial lifecycle is summarised in Fig. 1b.

## Part three: trial design and potential approaches for rare disease

Clinical trials in rare diseases present a distinct set of methodological and operational challenges that differentiate them markedly from trials in more common conditions. Foremost among these is the reality of extremely limited patient populations, which limits the feasibility of running multiple parallel trials. With only small numbers of eligible individuals available, often dispersed across regions or countries, trial design must prioritise efficiency, minimise waste of information, and adopt methods that maximise inferential power from every participant. Under these constraints, rigorous and thoughtful trial methodology becomes even more essential to avoid misleading conclusions and to ensure that promising therapies are not inappropriately abandoned, or ineffective therapies incorrectly adopted. Issues associated with rare paediatric CNS trials are faced by investigators of any rare cancer type, in which integrated and collaborative research is essential, and innovative and efficient designs with consideration of precision medicine are key [9]:

### Trial designs

Traditional trial structures, such as large parallel‑group randomised controlled designs, are impractical in rare settings. Instead, rare disease trials often rely on flexible and innovative methodologies tailored to small cohorts. Single‑arm phase II designs, including one‑stage or two‑stage approaches, remain widely used and can be highly effective when supported by robust sample size calculations. For example, a single‑arm design targeting a clinically meaningful response rate improvement (e.g., 35% vs. an assumed 20% for current therapies or a minimum effect) can provide adequate operating characteristics with only a few dozen participants, as long as the assumed ‘control’ rate is valid. Two‑stage designs enable early stopping for futility, offering ethical and logistical advantages by reducing participant exposure to ineffective treatments.

Adaptive designs, including response‑adaptive randomisation or multi‑arm multi‑stage structures, allow investigators to evaluate several therapeutic strategies simultaneously and to modify aspects of the trial as data accumulate. In rare diseases, where running multiple stand‑alone trials is rarely feasible, such designs could enable efficient evaluation of multiple interventions or treatment pathway components. They also support early termination of ineffective therapies, extension of recruitment when results remain inconclusive, and seamless addition of new trial arms. Nevertheless, implementation can be complex, and uptake within the rare disease community remains limited.

There are multiple phase I dose‑finding trials in rare paediatric or CNS tumours [10]. Traditional 3 + 3 designs have been most used, although these methods are increasingly recognised as statistically inefficient. Bayesian model‑based dose‑escalation methods, such as the Continual Reassessment Method (CRM) use all accumulated information to estimate dose–toxicity relationships more precisely [11]. However, in scenarios with only 2–3 dose levels, CRM may not always outperform simpler rules, and regulatory or statistical reviewers may require extensive justification of modelling assumptions. There is also the rolling 6 design (which aims to shorten trial duration compared to 3 + 3) and Bayesian optimal interval (BOIN) design (which aims to be more efficient than 3 + 3 and less complex than CRM).

Multi-arm trials, adaptive approaches, external control groups, and Bayesian methods offer solutions, but require specialised expertise, as do sophisticated methods of analysis (joint modelling, multi‑state survival, and longitudinal ordinal analysis).

### Control groups

The choice of therapy for a control group within a randomised trial is a key consideration. Although randomised trials are considered the best design, they can increase sample size requirements substantially, and patients or parents of children with poor prognosis cancers are sometimes reluctant to participate because they wish to have the new/experimental intervention—rather than a current standard of care therapy that has known limited efficacy. When populations are very small, external or synthetic control datasets derived from registries, natural history studies, or previous trials can provide an alternative, and the value and reliability of such approaches are being investigated. These datasets must be selected and analysed with great care, as differences in patient characteristics, outcome measurement methods, or follow‑up schedules, can introduce confounding and bias. Bayesian frameworks, for example, allow formal incorporation of external information through priors, supporting smaller sample sizes and more intuitive probabilistic interpretations. The SPRINT trial (selumetinib for children with inoperable plexiform neurofibromas) illustrates well how a single arm clinical trial in an uncommon tumour (50 patients) can be successfully supported by a real-world cohort study (92 patients) to show a substantial difference in progression-free survival. This evidence led to FDA Breakthrough and Orphan Drug approval, noting that the external control arm had good provenance, reliably measured outcomes, and patient characteristics were comparable to the trial [12].

### Outcome measures

Selection of primary and secondary endpoints are critical, especially for fit-for-filing trials. Surrogate endpoints have particular value for rare CNS tumour trials because trials are smaller and quicker than using ‘hard’ endpoints such as overall survival; changes to the ways that outcomes are defined and analysed potentially offer improvements in efficiency and statistical power. For instance, stroke trials have moved to ordinal outcomes [13], while in childhood cancer, the SPRINT trial used tumour response as the primary outcome [12], and Cheng et al showed that event-free survival is strongly associated with overall survival in genitourinary rhabdomyosarcoma [14]. Health-related quality of life (HRQoL), symptom burden, neurocognitive function and patient-reported outcome measures should be incorporated wherever feasible, including in early-phase studies. Such measures are particularly important in paediatric neuro-oncology, where treatment-related morbidity may substantially affect long-term functional outcomes and may help inform evaluation of clinical benefit beyond traditional toxicity and response measures. There are also issues over how endpoints are analysed. Standard approaches often reduce rich longitudinal tumour measurements to binary variables (response vs. no response) or rely solely on time to first event, discarding valuable longitudinal information. More efficient analytical approaches, such as augmented binary methods, joint models integrating tumour burden and survival outcomes, multi‑state models, or longitudinal ordinal approaches, can capture the full evolution of disease and improve power. These techniques are particularly valuable in rare disease trials, where maximising the information extracted from each participant is essential.

### Funder feedback on designs

Funders expect robust trial designs that can answer the research questions in rare cancers in a reasonable timeframe. Attendees provided examples of peer-review feedback following grant application submissions. These included: better justification of assumptions in modelling designs such as CRM, more details of translational research plans, clarity on the choice of surrogate endpoints, and consideration of interim futility analyses for potential early stopping. It was interesting to note that funders rarely asked for larger sample sizes and instead focused on re-designing elements.

In summary, our discussions highlighted trial designs for rare diseases must be highly efficient, flexible, and statistically rigorous. Leveraging adaptive and Bayesian methods, external data sources and advanced analytic approaches, these should generate robust evidence despite severe recruitment constraints, maximising the impact of every precious participant.

**Fig. 2:**
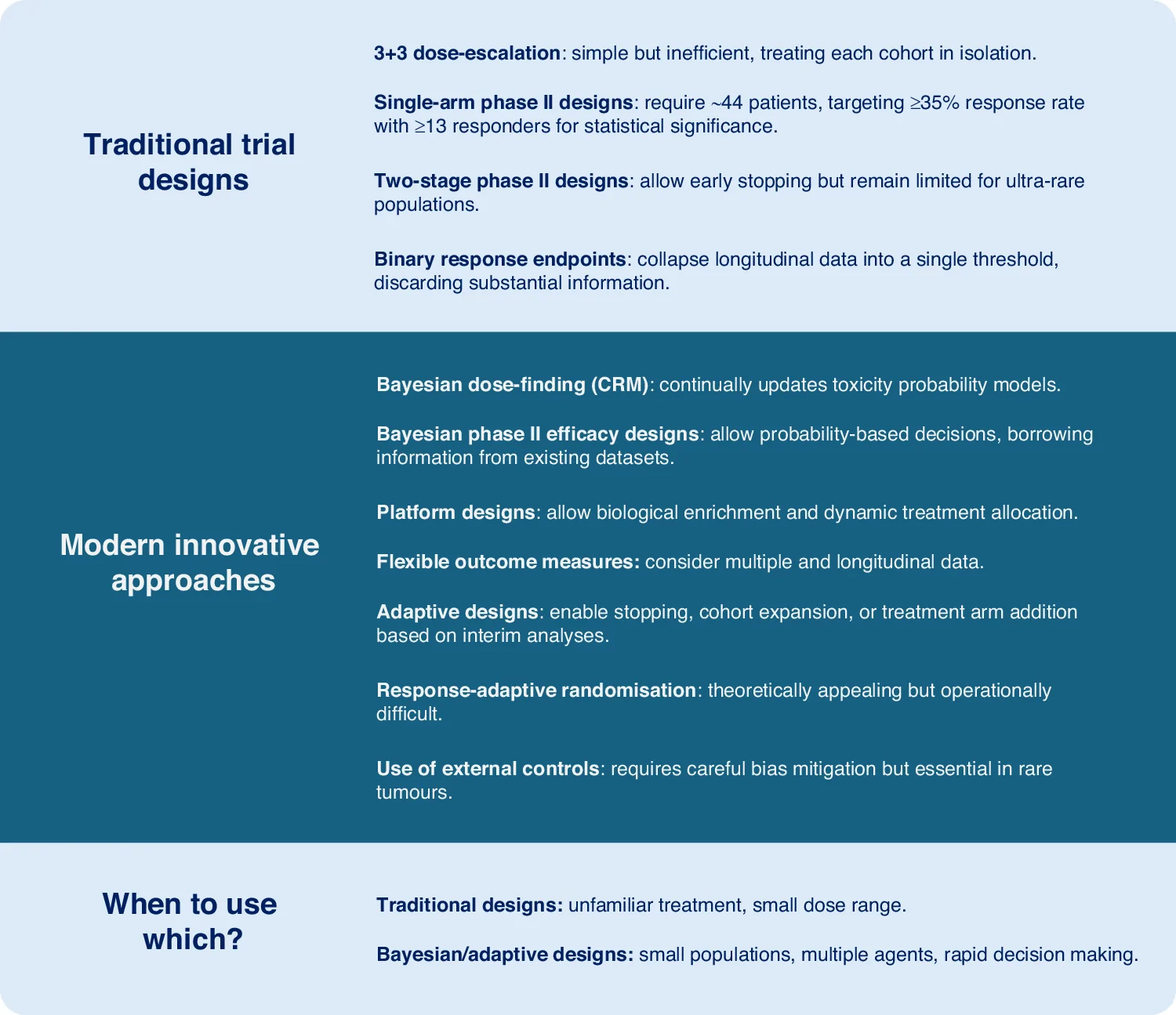
Summary of traditional vs. innovative trial designs for rare diseases

Key approaches to clinical trials in rare disease are summarised in Table 1B, alongside features of traditional and innovative statistical designs in Fig. 2.

## Part four: disease-specific strategies

The workshop next assessed the current status and future potential for clinical trial development in four key rare tumour type exemplars with significant unmet need: Craniopharyngioma, CPC, VHR-MB and REST with a particular focus on ETMR.

### Craniopharyngioma

Adamantinomatous craniopharyngioma (ACP) is a low incidence tumour (approximately 30 new UK cases in children and young people up to 25 years of age per year [4] with severe long-term morbidity and major endocrine and quality of life (QoL) consequences. ACP is frequently cystic in nature, and despite advances in hypothalamic sparing surgery and increased use of radiotherapy, around 25% of patients continue to have recurrence. Experience from the UK proton radiotherapy centres highlights there remains variation in surgical practice and timing of referral for radiotherapy across the UK [15–18]

Clinical trials—led by CONNECT and PNOC—have been developed, however, to date it has not been possible to open these trials in the UK, with a higher priority given to open other CONNECT and PNOC trials in the UK first.

The UK has been at the forefront of craniopharyngioma biology, with the development of genetically engineered mouse models, pre-clinical therapeutics and ‘omic studies of ACP trials [19–22]. Recent advances using BRAF/MEK inhibitors in papillary craniopharyngioma demonstrate clinical potential, including potential neoadjuvant and adjuvant opportunities for craniopharyngioma patients [23]. Novel agents, including MEK inhibitors, WNT pathway inhibitors, anti-inflammatory agents, and anti-VEGF targeted therapies, have emerging pre-clinical or early clinical signals [20, 23]. Imaging segmentation tools and cyst-focused strategies are also rapidly evolving [24].

Consensus emerged strongly that ACP represents the most immediately feasible opportunity for a UK‑led interventional trial in rare CNS tumours, with adequate patient numbers to support comprehensive initiatives. Key elements proposed included: (i) A UK initiated trial, focusing initially on progression pre- and post-radiotherapy and potential peri-radiotherapy windows, and (ii) Biologically-driven trial arms, specially focused on solid and/or cystic disease where appropriate, leveraging early phase access to novel agents or repurposing of existing agents. Radiology-driven endpoints (e.g., using automated segmentation and volumetric response in addition to the existing craniopharyngioma RAPNO criteria [24]) were considered important, alongside embedded biological sampling, especially of cystic fluid, to support mechanistic evaluation; experience in these is available in the UK [19] and wider networks [25]. Consideration of other relevant outcomes, including endocrine and patient reported outcomes, will be important within any trial pipeline, and early evidence of tumour efficacy was seen as critical. The group recognised the work to improve management of the consequences of craniopharyngioma through interventions such as anti-obesity drugs [e.g. Setmelanotide [26]].

There was universal agreement on the need to establish a national craniopharyngioma advisory multidisciplinary team (MDT), to support early clinical decision making [27] and support clinical trial recruitment.

### Choroid plexus carcinoma (CPC)

CPC is extraordinarily rare (3–6 UK cases/year) [4] and challenging due to its association with high-risk biology, poor outcomes, germline cancer predisposition (Li-Fraumeni syndrome(LFS)), and high treatment morbidity. Previous international trials have been limited by poor recruitment and resulted in a failure to assess significance [28, 29], highlighting the need for novel trial designs. Insufficient patient numbers preclude a UK/European trial and therefore global collaboration is essential. However prohibitively high trial costs in Europe (estimated at >€2.7 M for this trial at 2026 costs—representing costs to open multiple countries to recruit a rare disease) has resulted in the inability to secure a European sponsor for the upcoming North American-led PNOC trial (PNOC033), which consequently stopped UK and European participation [30]. Robust preclinical evidence for candidate agents is lacking, in part due to limited tissue availability, and learning from clinical experience has been limited by a lack of comprehensive data collection.

The Workshop consensus was that a new trial in the UK or Europe is not currently feasible, as current data and insufficient patient numbers do not support an alternative treatment strategy that differs significantly from the proposed PNOC trial, unless run as a parallel academic trial. A separate European sponsored trial using the same treatment concept and pooling the data for analysis may offer the opportunity to overcome the regulatory differences and contribute UK patients.

Critical actions were nonetheless identified, and the group agreed that efforts should be focused on a UK CPC service evaluation, participation in international registries such as the International SIOP-CPT Registry (www.uke.de/cpt) and sharing of biological samples to develop key datasets to support future trials. The potential to engage more broadly with tumour agnostic immunotherapy concepts/trials (e.g. B7-H3 targeted CAR-T) should also be explored, and CPC should be considered in inclusion criteria alongside other rare tumour entities, as part of platform trials or in biologically appropriate early phase trials.

### Very-high risk medulloblastoma (VHR-MB)

While medulloblastoma is the most common malignant brain tumour of childhood (approximately 60-65 new diagnoses per year in the UK) [4, 31], advances in its molecular subclassification have led to the recognition of smaller well-defined molecular groups with distinct clinical characteristics and outcomes [31, 32]. Specifically, Group3-*MYC* and SHH-*MYCN* carry the poorest prognoses (5-year progression-free survival <20%) and are essentially unresponsive to current multi-modal therapies. However, while new approaches are urgently required, these subgroups are rare (5–8 UK cases per year in total) and are underserved by current trials [4, 6]. Although the SIOP-HRMB clinical trial for high-risk medulloblastoma represents a major advance, the Group3-MYC and SHH-MYCN very-high-risk molecular subgroups are uncommon within the broader high-risk population and were not the primary focus of trial-specific therapeutic development [33].

The group reaffirmed the urgent need for new early‑phase capacity for *MYC/MYCN*‑driven medulloblastoma and related high‑risk subgroups including relapsed disease [34]. The workshop recognized that pre-clinical data are most advanced for the *MYC/MYCN* subgroups [35] (e.g. Aurora-A Kinase inhibition and checkpoint kinase (CHK1/2) inhibition [36, 37]), alongside the need to work across international partners, using innovative methodologies, to support meaningful conclusions and advance concepts based on the most efficient use of small patient numbers.

An emerging European ITCC phase 1/2 platform proposal was reviewed, which incorporates: A phase 1/2 component for all‑comers with relapsed embryonal brain tumours, mandated tissue and liquid biopsy to appraise circulating tumour DNA (ct-DNA), Bayesian dose‑finding, expansion cohorts, and potential combination strategies. A Phase 2 component which would, in the first instance, be based on highly-selected upfront *MYC/MYCN*‑amplified cohorts (infant and non‑infant), introducing targeted agents into established maintenance (non‑infant) or induction (infant) chemotherapy regimens [33, 38]. Importantly, the proposed trial design would also enable phase 2 expansion of patient-cohorts, not limited to VHR-MB, demonstrating early objective responses. As understanding of the role of liquid biopsy and clinical/survival correlates develops, future cohorts could include patients with evidence of residual disease post-therapy or early relapse, defined by CSF sampling and measurement of molecular biomarkers/ct-DNA [39].

This platform is intended to act as a pipeline for successive agents, including targets in other high-risk medulloblastoma groups such as *TP53* mutated SHH-MB and Group3-MB. Key strengths include preclinical candidates nearing readiness, strong engagement with ITCC, and enthusiastic UK support. Challenges include drug availability, harmonisation with SIOPE late-phase trials, engaging the patient/parent community on acceptability of trial concepts which include proposed changes to upfront therapies, and operational complexity. Ongoing discussions within the relevant European collaborative groups (SIOPE, ITCC) are addressing harmonisation of future studies in relapsed disease.

### Rare embryonal and sarcomatous tumours (REST) and embryonal tumours with multilayered rosettes (ETMR)

In the molecular era, re-classification of paediatric brain tumours has led to the recognition of several novel entities under the umbrella of REST with distinct genetic, histopathological, and clinical characteristics; ETMR is a prominent example [40]. Historically, REST were often treated uniformly, with treatment extrapolated from other embyonal tumours such as medulloblastoma, and so prospective data is required to establish optimal therapeutic approaches especially as many of these tumours have poor outcomes. A European REST registry (funded by The Brain Tumour Charity; currently in set-up) aims to collect data and biological samples. Challenges include absence of uniform management strategies to allow comparison of outcome data and ensuring optimal patient recruitment and collection of samples across Europe.

ETMR is among the rarest and most lethal paediatric brain tumours (approximately 20 cases per year in Europe; 1–3 per year in the UK; 25% 5-year overall survival) [5]. Three international initiatives—PNOC, CONNECT, and SIOPE—have developed different trial concepts with additional challenges with respect to alignment. The PNOC group has now been joined by the SIOPE REST Group, including UK leaders, that have led to improvements in trial design, including randomisation of therapies and more standardised treatment pathways.

The workshop was in consensus that a global trial is required, but opening PNOC trials in Europe is extremely difficult due to sponsorship, regulatory, and data transfer barriers. A parallel European academic trial, potentially led by CRCTU, could be feasible pending statistical review but issues remain regarding funding a single disease trial with multiple sites, each expected to recruit only one to two patients.

A REST platform trial which allows recruitment of several REST entities may be a future vision but requires development of robust trial questions and designs for each tumour type. The European REST registry together with other similar initiatives may offer the greatest immediate benefit in building prospective datasets to learn from, as well as providing real-life controls for future trials.

**Fig. 3:**
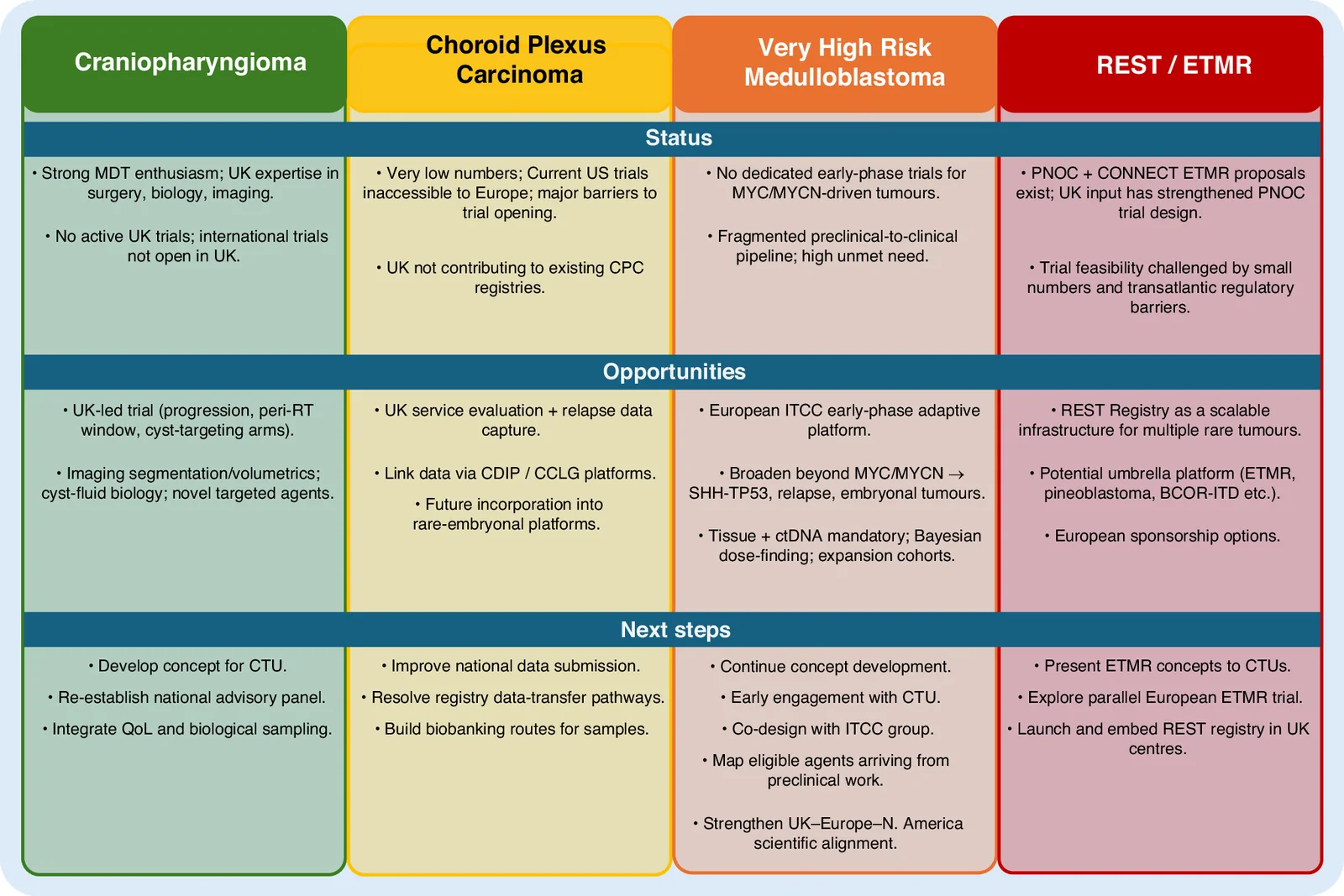
Rare childhood CNS tumour clinical trials in key exemplar tumour types: Status, opportunities and next steps

Key outputs from Session Four are summarised in Fig. 3.

## Part five: cross-cutting strategic themes and outputs—a roadmap for rare childhood CNS tumours

Finally, the group focussed on (i) identification of critical cross-cutting themes which will be essential to progress across all tumour groups, and (ii) convergence on specific high-priority strategic directions, as the major outputs from this workshop (Table 1, Fig. 3):

### National coordination

Establish a UK rare CNS tumour research strategy integrating registry development, trial feasibility mapping, biological data pipelines, and PPIE structures, coordinated by the UK3CR Children’s CRG CNS Tumour Subgroup.

### International connectivity

Active engagement and leadership within organisations including European, North American (SIOPE, ITCC, PNOC, CONNECT) and related international networks is essential, as no single country can effectively develop evidence independently for ultra-rare childhood CNS tumours. UK researchers maintain strong representation within these organisations. Strengthening these links—and those to other consortia including emerging partners in Asia and beyond—was considered crucial, particularly given the high frequency of trans-Atlantic trials among rare tumour types.

### The principle of “Learning from Every Patient”

Participants emphasised that meaningful progress in rare tumours requires capturing clinical, biological, radiological, and quality of survival data from all patients, not solely those enrolled in trials. This principle should underpin discussions on trials, registries, analytics, and multi-centre data-sharing frameworks.

### Platform trials and advisory structures

UK-led platform trials should be developed where feasible (e.g., craniopharyngioma, VHR-MB) and functioning National advisory groups ensured for clinical decision-making, building on the UK’s success with a national ependymoma panel (the Ependymoma Multidisciplinary Advisory Group (EMAG)) [27], to support local decision making, with craniopharyngioma as an immediate priority. These were seen as essential for standardising care and supporting trial readiness in all disease groups discussed.

### Strengthen preclinical-to-clinical pipelines

Ensure translational science (including small molecule and immunotherapy development) is supported, undertaken to standards of evidence required to support clinical translation, and rapidly integrated into trial frameworks.

### Importance of biology and imaging

Many proposed trials integrate systematic collection of tissue, cyst fluid, and liquid biopsies, alongside AI‑enabled imaging metrics. This was recognised as essential both for mechanistic insight and endpoint optimisation.

### Trial infrastructure and funding challenges

International trials face hurdles in drug procurement, distribution, and divergent regulatory frameworks. A UK‑ or Europe‑led model may unlock more realistic routes for rare‑tumour trials. Funding was identified as a cross-cutting determinant of success. Registries, biological sample collection, database infrastructures, advisory panels and clinical trials all require dedicated and sustainable resources. Early engagement with funders to establish rare tumour-specific funding streams was therefore viewed as an essential component of the roadmap.

### Integration with national structures

The group recognised the need for tighter integration with our National body, CCLG, for governance, funding alignment, and registry coordination. Strategic dialogue is underway between CCLG leadership and workshop organisers to embed rare childhood CNS tumour priorities within national frameworks. Continued leverage of, and integration within, the National Institute of Health and Care Research (NIHR)/Experimental Cancer Medicine Centre paediatric network (ECMC; www.ecmcnetwork.org.uk)—a UK-wide collaboration of 12 specialist centres dedicated to running early-phase clinical trials for children and young people with cancer—is essential.

### National registries and data infrastructure

Across all tumour groups, the absence of systematic UK data collection was repeatedly identified as a fundamental barrier. Obstacles to this which were identified included the need for data and material transfer agreements (DTAs, MTAs), contract negotiations, information governance approvals and the need to develop long-term funding and infrastructure support. Initial experience with multi-centre data collection for medulloblastoma in the UK, and as part of international studies, has enabled assessment of the performance of clinical protocols in the ‘real-world’ setting [41–43]. Several proposals emerged including: use of CCLG-hosted shared data platforms; alignment with the European REST registry; leveraging NIHR/ECMC data infrastructures for relapse cases, and linking national biological sample collection initiatives (e.g. the Stratified Medicine Paediatrics study (SMPaeds [44]) and the National VIVO biobank for childhood and young people’s cancer biomaterials (www.vivobiobank.org)) to clinical datasets. Across CPC, REST, and craniopharyngioma, significant enthusiasm exists for shared UK data platforms (e.g. via. CCLG or hybrid approaches, such as the ECMC Children and Young Persons (CYP) Cancer Data Integration Platform). A simple, low‑burden submission mechanism would be essential for centre compliance.

### Registry-first approach for ultra-rare tumours

For ETMR, CPC, and other ultra-rare groups, there is a need to prioritise registries and biological data capture while preparing for future trials.

### PPIE

A national PPIE panel for rare CNS tumours was proposed and strongly supported.

### Early-career researcher (ECR) development

The group identified ECR involvement as essential for long-term sustainability. A plan was proposed to pair senior UK3CR members and triallists with ECRs and to establish a joint working group dedicated to training and succession planning.

### Strategic funding and sustainability

Participants recognised that sustainable funding will be a critical enabler of progress across all major priorities, including registries, biobanking, translational research, advisory structures and clinical trials. However, many opportunities identified during the workshop can build upon existing national and international infrastructures, including UK3CR, CCLG, NIHR/ECMC networks, established biobanking initiatives and international research consortia. A strategic approach is therefore required that combines alignment of existing resources with targeted new investment from charitable funders, governmental agencies, international partnerships and industry collaborators. Development of high-quality prospective registries and biological datasets was viewed as a particularly important near-term priority, both to accelerate scientific discovery and to strengthen the evidence base required for future funding applications and interventional studies. Long-term success will depend upon embedding rare childhood CNS tumour research within sustainable national and international funding frameworks.

Against these universal strategic principles, immediate priorities identified were: (i) Developing a UK-led craniopharyngioma trial, (ii) Progressing a VHR-MB platform with ITCC partners, (iii) Activating the REST registry and strengthening UK trial-readiness for ETMR, and (iv) Expanding CPC data collection (Fig. 3). Early engagement with CTUs, PPIE, and stakeholders in concept development will be essential, alongside embedding modern statistical design and ‘fit-for-filing’ principles across all initiatives.

## Conclusions and outlook

We have established an essential roadmap for a strategic programme in rare paediatric CNS tumour clinical research, centred on platform‑based trial innovation, stronger international alignment, and learning from every patient to accelerate collective therapeutic progress. While major barriers remain, there is also unprecedented opportunity to advance therapies and outlook for these rare tumour groups. Immediate next steps include establishment and/or strengthened support of dedicated working groups for craniopharyngioma, CPC, VHR-MB and REST, engagement with UK and international partners to develop registry and trial concepts, integration of these priorities within UK3CR and CCLG structures, and review of progress through future stakeholder workshops. Together, these outputs lay the foundation for participation and leadership in this global effort.

## Data Availability

This manuscript does not contain any foundational data.
